# Treating loss-to-follow-up as a missing data problem: a case study using a longitudinal cohort of HIV-infected patients in Haiti

**DOI:** 10.1186/s12889-018-6115-0

**Published:** 2018-11-19

**Authors:** Deanna P. Jannat-Khah, Michelle Unterbrink, Margaret McNairy, Samuel Pierre, Dan W. Fitzgerald, Jean Pape, Arthur Evans

**Affiliations:** 1 Division of General Internal Medicine, Department of Medicine, Weill Cornell Medical College, 1300 York Avenue, New York, NY USA; 2 Center for Global Health, Weill Cornell Medicine, New York, USA; 3 Haitian Group for the Study of Kaposi’s Sarcoma and Opportunistic Infections (GHESKIO), Port au Prince, Haiti

**Keywords:** HIV, AIDS, Kaplan Meier, Complete case, Multiple imputation, Inverse probability weights

## Abstract

**Background:**

HIV programs are often assessed by the proportion of patients who are alive and retained in care; however some patients are categorized as lost to follow-up (LTF) and have unknown vital status. LTF is not an outcome but a mixed category of patients who have undocumented death, transfer and disengagement from care. Estimating vital status (dead versus alive) among this category is critical for survival analyses and program evaluation.

**Methods:**

We used three methods to estimate survival in the cohort and to ascertain factors associated with death among the first cohort of HIV positive patients to receive antiretroviral therapy in Haiti: complete case (CC) (drops missing), Inverse Probability Weights (IPW) (uses tracking data) and Multiple Imputation with Chained Equations (MICE) (imputes missing data). Logistic regression was used to calculate odds ratios and 95% confidence intervals for adjusted models for death at 10 years. The logistic regression models controlled for sex, age, severe poverty (living on <$1 USD per day), Port-au-Prince residence and baseline clinical characteristics of weight, CD4, WHO stage and tuberculosis diagnosis.

**Results:**

Age, severe poverty, baseline weight and WHO stage were statistically significant predictors of AIDS related mortality across all models. Gender was only statistically significant in the MICE model but had at least a 10% difference in odds ratios across all models.

**Conclusion:**

Each of these methods had different assumptions and differed in the number of observations included due to how missing values were addressed. We found MICE to be most robust in predicting survival status as it allowed us to impute missing data so that we had the maximum number of observations to perform regression analyses. MICE also provides a complementary alternative for estimating survival among patients with unassigned vital status. Additionally, the results were easier to interpret, less likely to be biased and provided an alternative to a problem that is often commented upon in the extant literature.

**Electronic supplementary material:**

The online version of this article (10.1186/s12889-018-6115-0) contains supplementary material, which is available to authorized users.

## Background

HIV programs are often assessed by the proportion of patients who are alive and retained in care, which has direct consequences for funding and programmatic services offered [1, 2]. However, among individuals who initiate antiretroviral treatment (ART), the reported rate of lost to follow up ranges from 5 to 53% [1, 3–10]. Clinically, these LTF patients are at risk for adverse outcomes such as medication resistance, transmission to others, lack of care, or at best, incomplete medical records when they transfer care to another clinic [1, 6, 7]. Programmatically, lost to follow up leads to underestimates of retention which could be mis-interpreted as under-performance on program outcomes [1, 5, 6, 11].

The category of lost to follow-up (LTF) is not a homogeneous outcome—e.g., “dead” or “alive”—but rather a heterogeneous category of three disparate health states: undocumented deaths, undocumented or silent transfers to another source of HIV care, or alive and complete disengagement from HIV care [12–14]. Alive and being retained in care is synonymous with the proportion of patients who are neither dead nor LTF. The fact that LTF is part of the definition makes this outcome complex and problematic.

In reality, LTF is a marker for missing data on vital status. We argue that LTF should not be treated as a legitimate outcome category because it’s meaning can easily change over time and across sites. For example, patients who silently transfer to another provider, move domiciles or die outside of a healthcare facility could all be classified as LTF. Thus, studying predictors of LTF should be avoided. Instead, LTF should be considered a missing data problem that needs to be solved. We present a unique application of MICE to impute both missing outcome (vital status) and missing covariates, simultaneously, using a large longitudinal cohort of patients from Haiti who were treated for HIV infection, and compare the results with MICE to the more traditional analytic methods of using complete cases and inverse probability weights. We also evaluated associations that were predictive of death using three different methods: complete case, inverse probability weights and multiple imputation with chained equations.

### Statistical methods for handling missing vital status

In the HIV literature, for studies assessing predictors of mortality/survival, the most common methods of dealing with LTF are complete case analysis, survival models that censor those LTF, and tracing with inverse probability weights [10, 15–22]. But there are other methods, including simple imputation, multiple imputation, and Bayesian analysis [15]. Each method has different underlying assumptions about the missing data.

#### Complete case analysis

Complete case analysis omits observations with missing data in multivariable analyses. It is the default method, employed automatically, of most statistical software programs. As only complete observations are used, sample size is decreased, statistical power is compromised, and study results are often biased [10, 16].

#### Kaplan Meier survival analysis

Kaplan Meier analysis assumes that lost to follow up is unrelated to mortality. To state this another way, patients who are censored due to LTF have the same probability of survival as those who are not lost to follow up [23]. However, one cannot verify the Kaplan Meier assumption without more information. From the extant literature, studies have traced patients who are categorized as lost and found that between 12 and 87% were dead [24]. With this wide range in mind, it is impossible to say if LTF is associated with higher mortality, lower mortality, or if there is no association. Employing this method, patients who are LTF are censored at a time point typically defined by the date when vital status was last verified. It is often used for analyzing HIV cohort data because all cases can be included, at least for the duration that they were followed before being lost.

#### Inverse probability weights from tracing

Inverse probability weights (IPW) offer another general method for dealing with missing data [17–21, 25]. In the HIV literature, they are often used in conjunction with tracing data. This approach involves using physical or contact tracing to determine the true vital status among a sample of those LTF [20–22, 25]. Then, assuming this sample is representative of all LTF, tracing data is used to apply weights to the subjects with no missing outcome data, so that the weighted analysis provides less biased results, compared to the biased results when using (unweighted) complete cases. The results of the tracing are used to calculate the inverse probability of being a complete case (given the unique set of patient characteristics, including predictors and outcomes), which is used to weight each of the complete cases [20–22, 25]. This method assumes that those who are unsuccessfully traced have a mortality that can be accurately estimated from those successfully traced.

For example, consider a simple analysis to assess whether gender predicts mortality. Among 100 women 50 are documented dead and 50 are documented alive, among 100 men there are 20 documented dead, 20 documented alive, and 60 LTF. A “complete case” analysis suggests that men and women have the same risk of dying (RR = 1), since 50% of the men died and 50% of the women died. However, suppose all 60 of the men LTF were successfully traced and found to be dead. For women who died, all were complete cases, so the IPW is the inverse of the probability of being a complete case, or 1/1.0, or 1. For all women who did not die, all were also complete cases, so the IPW is also 1/1.0. For men who were alive, all were complete cases, so their IPW is also 1/1.0. But for men who died (*n* = 80, 20 complete case deaths and 60 traced deaths), the probability of being a complete case was 20/(20 + 60), and therefore the IPW is 1/.25, or 4. If we apply these weights and do an IPW analysis—giving complete case men who died 4x the weight of any other complete case—then the average mortality among men is 20 × 4/(20 + 20 × 4) = 80%; and the risk of dying among men compared to women is 80/50 = 1.6.

Note: If only a fraction *(f)* of the LTF get traced, then each of the traced cases is weighted by the inverse probability of being traced, that is, by 1/*f*.

However the performance of the IPW model is dependent on methods used to track patients. In resource-limited settings, tracing is difficult, costly, and often unsuccessful. In our case study, Haiti does not have a unique national identification number for its citizens, making it difficult to track patients across various health systems or to verify vital status by referencing a current national death registry [3].

#### Multiple imputation with chained equations (MICE)

Multiple Imputation with Chained Equations (MICE) is a less commonly used method for estimating the vital status of those LTF. Although MICE is commonly used to impute missing covariate (predictor) data, [10, 26, 27] it can also be used to impute missing outcome data [26, 27]. MICE is optimal when less than 30% of a variable’s data are missing and when subjects with missing data are only randomly different (“missing at random”) from those subjects who share an identical set of patient characteristics, or covariate values [28–31]. However, to our knowledge, no articles in the extant HIV literature have reported results after imputing both the outcome and covariates simultaneously.

The aim of this analysis is to present the application of MICE to impute both missing outcome (vital status) and missing covariates, simultaneously, using a large longitudinal cohort of patients from Haiti who were treated for HIV infection, and compare the results with MICE to the more traditional methods of using complete cases, survival analysis and inverse probability weights. Specifically, we compare adjusted logistic regression models for factors associated with death using complete case, IPW and MICE.

## Methods

### Study population

The study population is a cohort of 910 individuals age 13 years or older who initiated antiretroviral therapy (ART) for HIV according to international guidelines between March 2003 and April 2004 in Haiti [32, 33]. The cohort was followed for ten years through 2015. Details of this cohort are described in previous publications [32, 33].

### Clinical measurements and outcomes

Clinical characteristics available from routinely documented data included body weight, CD4+ cell count (CD4), WHO stage, and diagnosis of tuberculosis. Sociodemographic data included age, sex, severe poverty, and residence within the city of Port au Prince. Severe poverty was defined as living on less than one United States dollar per day. Date of death and transfer were documented in the medical record. Lost to follow-up was defined as no documented death or transfer and no clinical visit or pharmacy pick-up during the last 180 days of the 10-year follow-up. Patients who were classified as LTF were traced by clinic staff at the time of their 10-year anniversary to ascertain vital status.

### Missing data

The frequency of missing data at baseline was 3% for weight, 12% for CD4 count, and 12% for vital status at 10 years of follow-up. The 71 subjects who were documented to have transferred their care to another clinic (8%) were assumed to be alive at 10 years.

### Multiple imputation with chained equations (MICE)

Data were assumed to be missing at random; i.e. considered only randomly different from other subjects that share the same pattern of values for the non-missing variables. MICE was used to impute all missing values, whether for missing covariates, such as CD4 count and weight, or for missing vital status (LTF) at 10 years of follow-up. We used Stata’s implementation of MICE, which allows the imputation of various types of variables (categorical, ordinal, or continuous) in chained equations using a semi-Bayesian approach in [30] In this study, CD4 and baseline weight were continuous variables and vital status was a dichotomous variable. Results from multivariable fractional polynomial models on complete case data indicated that CD4 is best represented as a cubic function and baseline weight is best represented as a squared function. These transformations were included in the multiple imputation model. Equations were created to impute missing values and were composed of all variables used in the fully adjusted models [30]. Predictive mean matching using 5 nearest neighbors was used to impute CD4 and baseline weight [34–37]. Twenty imputations were computed based on current guidelines in the literature [30]. Various diagnostic measures were performed to check the fitness of the generated datasets. Specifically, proportions were calculated to assess imputed values of categorical variables and continuous variables were assessed using trace plots [38]. The Stata command *midiagplots* was used to assess the imputed datasets [38].

### Classification and regression trees (CART)

Classification and regression trees were utilized to ascertain if any interaction should be incorporated into the multiple imputation [39]. Classification trees, in contrast to traditional statistical models, are especially useful for assessing for interactions when there are significant amounts of missing data [40]. After building the tree and pruning it using the R command *cptable*, no statistically significant interactions were found [41].

### Statistical analysis

#### Kaplan Meier

Survival estimates were calculated using Kaplan Meier analyses and a Kaplan Meier curve was generated. Time from enrollment to death or end of study censor (ten years after enrollment with a maximum date of June 26, 2014) was calculated. Participants who were classified as LTF were censored at their last visit to the clinic.

#### Inverse probability weights from tracing

In September 2013, staff attempted to contact all 156 patients who were classified as LTF, using telephone and home visits. Results of this tracing method were used to create inverse probability weights (IPW) that were applied to cases with similar covariates and known vital status.

#### Multiple imputation with chained equations

The mi suite of commands from STATA was used to perform analyses using the multiply imputed datasets. Stata’s mi suite of commands follows Rubin’s rules for the combination of results across imputed datasets [42].

#### Logistic regression

For each predictor (covariate), logistic regression models were created to calculate odds ratios and 95% confidence intervals for being dead after 10 years of follow-up (univariable models). Additionally, we created multivariable (fully adjusted) models that included all clinical and sociodemographic variables. Although age, weight, and CD4 count were measured as continuous variables, when reporting the results of the logistic regression models, we describe the effects of a 10-year age difference, 10-kg weight difference, and 100-cell difference in CD4 count.

#### Sensitivity analysis — Multiple imputation then deletion

As a sensitivity analysis, we performed multiple imputation of all missing data, followed by deletion of all cases of missing outcomes. In this method, both the outcome and covariates are imputed and after the datasets are created, observations where the outcome was imputed are deleted from the dataset running the same univariable and multivariable models [43]. This method has been reported to lead to more efficient estimates and narrower confidence intervals [43].

All analyses were performed using STATA version 13 and R version 3.4.2. Additional file 1.

#### Ethics and consent to participate

The institutional review boards at GHESKIO and at Weill Medical College of Cornell University approved this analysis.

## Results

### Outcome tracing

Among the 156 patients who were categorized as LTF, the clinical team was able to trace and find 45 (29%). Of the 45 patients successfully traced, 37 (82%) were found to be alive and 8 (18%) had died prior to 10 years of follow-up. Based on the 18% risk of death among those successfully traced, we assume that 18% of the 156 LTF (*n* = 28) were dead at 10 years and the remainder were alive. Since the probability of being known alive at 10 years among all patients who were actually alive (known alive plus number estimated to be alive among LTF by the tracing method) is 0.79, then the IPW for all those subjects who are known alive is 1/0.79.

### Missing data/ diagnostics of the multiple imputation

**Fig. 1:**
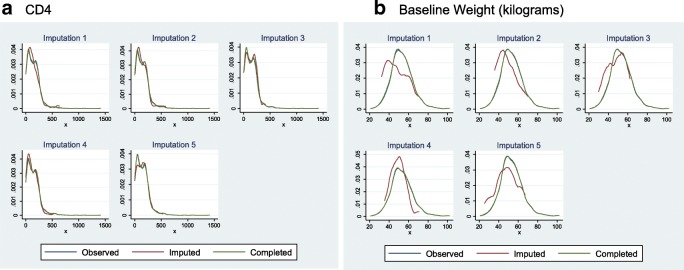
Kernel Density plots for imputed CD4 and Weight for first five imputed datasets. Panel **a** shows the plots specific to the imputation of the CD4 variable. Panel **b** shows the plots specific to the imputation of the Weight variable

**Fig. 2:**
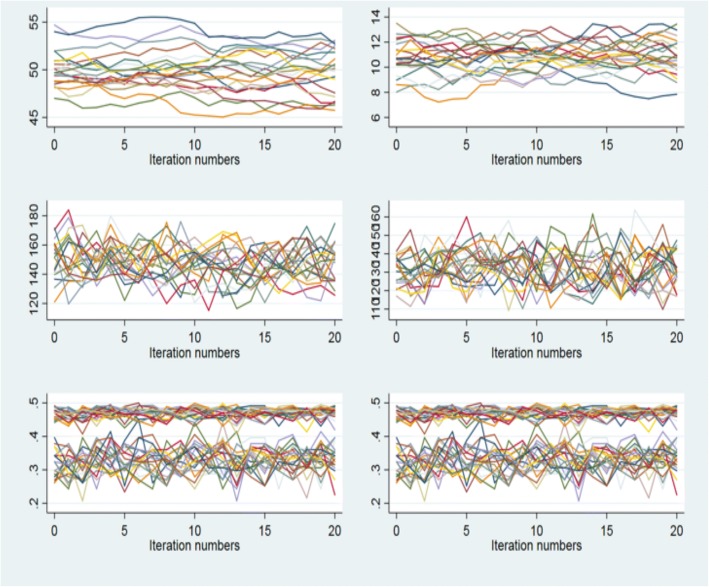
Trace Plots of imputed data across 20 imputed datasets

Convergence was achieved when MICE was performed. To assess the results of the multiple imputation, kernel density and trace plots were constructed. The kernel density plots for the imputed values of CD4 and weight are shown in Fig. 1 for the first 5 imputed datasets. The means and interquartile ranges for CD4 and weight are similar to the observed non-missing observations in the dataset (Table 1). Figure 2 displays the trace plots for the twenty imputed datasets. These plots show no discernable pattern, which is the result expected of a well-executed multiple imputation.

**Table 1:** Comparison of CD4+ and weight using Complete Case, Imputation and Imputation then Deletion

Clinical Characteristic	Without Imputation	With Imputation	With Imputation then Deletion
CD4+ count (cells/μL)			
Median (IQR) [range]	131 (51–212) [0–1400]	141 (60–223) [1–1416]	124 (53–138) [1–1416]
Missing	12%	N/A	N/A
Body weight (kg)			
Men median (IQR)	56 (50–63)	55 (48–62)	55(48–62)
Women median (IQR)	49 (44–56)	48 (42–54)	48 (42–55)
Missing	3%	N/A	N/A
Outcome	12%	N/A	12%

### Probability of death after 10 years of follow-up using Kaplan Meier, IPW and MICE: A comparison

**Fig. 3:**
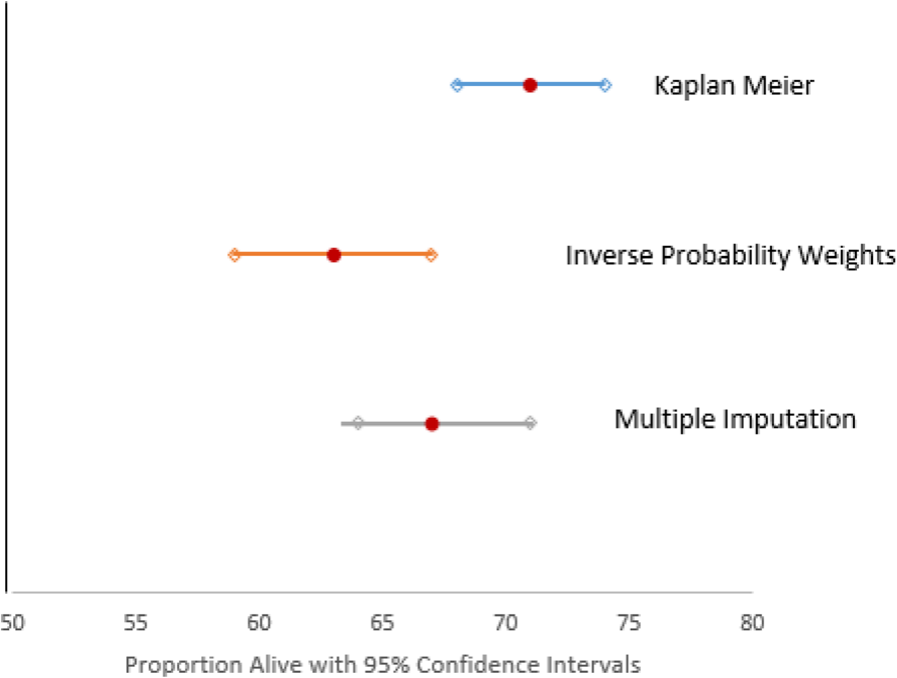
Survival estimates from Kaplan-Meier, IPW, and MICE

At 10 years, and accounting for the tracing efforts described above, 53% of patients (*N* = 482) were alive and engaged in care, 27% (*N* = 246) were confirmed dead, 12% (*N* = 111) were LTF, and 8% (*N* = 71) had transferred to another clinic for care. Survival was ascertained to be 71% (95% CI: 68–74%) by Kaplan Meier, 63% (95% CI: 59–67%) by IPW, and 67% (95% CI: 64–71%) by MICE (Fig. 3) [44].

### Predictors of death using complete case, IPW and MICE: A comparison

The weighted sample when using IPW weights from tracing had 111 fewer observations (*N* = 799) compared to the MICE dataset, which included all observations (*N* = 910), because any subject with missing covariate data was dropped. The complete case model should have the least number of observations (*N* = 735) because any case with any missing value was dropped from the analysis.

Table 2 displays the logistic regression results for each individual predictor of death using three types of models: complete case (CC), inverse probability weighting (IPW) and multiple imputation with chained equations (MICE). Severe poverty was statistically significant across all models and the odds ratio had an approximate 20% difference between CC and IPW (CC OR = 1.78, IPW OR = 1.59, MICE OR = 1.74). WHO stage and baseline weight were statistically significant across all models and had similar odds ratios from the three methods (Table 2). CD4 had a similar point estimate across all 3 models (CC OR = 0.86, IPW OR = 0.86, MICE OR = 0.85). However, the point estimate was not statistically significant in the CC model. Age was slightly different across all three models (CC OR = 1.17, IPW OR = 1.26, MICE OR = 1.20). Similar to CD4, age was not statistically significant for CC. Baseline tuberculosis was statistically significant across all models and had a slight variation in the point estimates (CC OR = 1.97, IPW OR = 1.92, MICE OR = 1.98). Gender and residence were not statistically significant in any model.

**Table 2:** Comparing Predictors of Death using Inverse Probability Weighting from Tracing and Imputation^a^

	Complete Case OR (95% CI)	IPW OR (95% CI)	MICE OR (95% CI)	MICE with deletion OR (95% CI)
	*N*=735^b^	*N*=799^2^	*N* = 910	*N* = 799
Female	0.83 (0.61, 1.13)	0.79 (0.57, 1.10)	0.83 (0.62, 1.12)	0.81 (0.60, 1.10)
Age (for 10-yr difference)	1.17 (1.00, 1.37)	**1.26 (1.06, 1.51)**	**1.20 (1.03, 1.39)**	**1.20 (1.03, 1.40)**
Residence	1.13 (0.81, 1.56)	1.28 (0.90, 1.82)	1.18 (0.87, 1.60)	1.17 (0.85, 1.61)
Severe poverty	**1.78 (1.2, 2.44)**	**1.59 (1.14, 2.24)**	**1.74 (1.27, 2.37)**	**1.71 (1.25, 2.34)**
CD4 (for 100-cell difference)	0.86 (0.74, 1.00)	**0.86 (0.75, 0.99)**	**0.85 (0.73, 0.98)**	**0.84 (0.72, 0.98)**
Baseline Weight (for 10-kg difference)	**0.67 (0.58, 0.79)**	**0.69 (0.56, 0.83)**	**0.65 (0.56, 0.76)**	**0.66 (0.57, 0.77)**
WHO stage	**2.19 (1.60, 3.00)**	**2.19 (1.56, 3.06)**	**2.18 (1.62, 2.95)**	**2.19 (1.61, 2.98)**
Baseline Tuberculosis	**1.97 (1.20, 3.22)**	**1.92 (1.13, 3.25)**	**1.98 (1.23, 3.18)**	**1.95 (1.21, 3.15)**

^a^Models were constructed with only one covariate

^b^For the CD4 only model *N* = 671; Baseline weight model *N* = 671

^2^For the CD4 only model *N* = 710

***p*** **-value ≤ 0.05**

Although in univariable analysis (single predictor), the beta coefficients have similar point estimates regardless of method; differences are seen among the point estimates in multivariable models. Table 3 displays results from multivariable logistic regression models using the three methods. Severe poverty was statistically significant across all models and the odds ratio had an approximate 10% difference between CC and MICE (CC OR = 1.63, IPW OR = 1.64, MICE OR = 1.80). Similarly, WHO stage was statistically significant across all models and had an approximate 15% difference between the odds ratios from the CC models (OR = 1.50) compared to the MICE model (OR = 1.76). Age and baseline weight were statistically significant across all the models with a slight variation in the point estimates and 95% confidence intervals. Female gender was found to be protective for death across all three models; however, it was statistically significant only in the MICE model (OR 0.62; 95% CI: 0.44–0.87) and there was about a 10% difference between the IPW and the MICE models’ odds ratios. Baseline tuberculosis infection was associated with a higher odds of death across the three models, however it was only statistically significant in the complete case model (OR 1.83; 95% CI: 1.05–3.20). Additionally, there was an approximate 20% difference between the odds ratios of the CC and the MICE models for baseline tuberculosis infection. Port au Prince residence and CD4 were not statistically significant across the three models.

**Table 3:** Comparing Predictors of Death using Inverse Probability Weighting and Imputation in adjusted models

	Complete Case OR (95% CI)	IPW OR (95% CI)	MICE OR (95% CI)	MICE with deletion OR (95% CI)
	*N* = 660	*N* = 698	*N* = 910	*N* = 799
Female	0.74 (0.51, 1.08)	0.69 (0.46, 1.03)	**0.62 (0.44, 0.87)**	**0.63 (0.45, 0.88)**
Age (per 10 yrs)	**1.34 (1.11, 1.60)**	**1.44 (1.18, 1.76)**	**1.37 (1.17, 1.61)**	**1.37 (1.16, 1.61)**
Residence	1.08 (0.74, 1.56)	1.38 (0.93, 2.05)	1.18 (0.85, 1.65)	1.18 (0.84, 1.64)
Severe poverty	**1.63 (1.12, 2.36)**	**1.64 (1.10, 2.44)**	**1.80 (1.28, 2.52)**	**1.76 (1.25, 2.47)**
CD4 (per 100 cells)	0.95 (0.82, 1.09)	0.92 (0.81,1.05)	0.91 (0.80, 1.05)	0.92 (0.80, 1.06)
Baseline Weight (per 10 kg)	**0.70 (0.58, 0.85)**	**0.69 (0.54, 0.87)**	**0.66 (0.55, 0.78)**	**0.67 (0.56, 0.80)**
WHO stage	**1.50 (1.04, 2.17)**	**1.62 (1.10, 2.40)**	**1.76 (1.25, 2.47)**	**1.77 (1.28, 2.47)**
Baseline Tuberculosis	**1.83 (1.05, 3.20)**	1.78 (0.97, 3.26)	1.61 (0.97, 2.67)	1.61 (0.97, 2.68)

***p*-value** **≤ 0.05**

### Sensitivity analysis

Results from the sensitivity analysis were very similar to the results from the MICE models for univariable and multivariable models. For severe poverty and baseline weight, with the multivariable model only, the 95% confidence intervals were slightly narrower for the MICE with deletion compared to the MICE models without deleting cases with imputed outcomes (Table 2).

## Discussion

Among the first cohort of HIV patients who initiated antiretroviral therapy in Haiti from 2003 to 2014, we aimed to find associations that were predictive of death using three different methods: complete case, inverse probability weights and multiple imputation with chained equations. These three procedures have different assumptions and differed in the number of observations included in the adjusted model due to how missing values for co-variates were addressed. Although the point estimates were similar across the three models, for statistically significant factors we found as much as a 20% difference in odds ratio values. For statistically significant factors, such as severe poverty and WHO stage, the odds ratios in the MICE models were farther away from the null compared to the CC and IPW models. Severe poverty was a statistically significant predictor of death in the MICE model (OR 1.80; 95% CI: 1.28–2.52). In a similar cohort from the same clinic in Haiti, income was associated with a higher odds of attrition (OR 1.65; 95% CI: 1.25–2.19) [45]. Additionally, these estimates are similar to those from an intensive contact tracing program performed in Malawi on HIV positive patients, which found about 70% of people who were initially categorized as LTF were alive and 30% were dead [13].

Worldwide, LTF rates for patients who have initiated ART treatment for at least one year range from 5 to 53% [1, 3–10]﻿. Patient characteristics associated with becoming LTF include being clinically ill, as measured by CD4 count or WHO symptom staging, low socioeconomic status, and concern for stigma, as well as structural factors such as transportation issues [3, 7–10, 45–47]. Several studies have reported high rates of re-engagement in care by patients who were previously labeled as LTF [3, 4, 7, 8, 11, 45]. A study in South Africa found that up to 50% of patients who disengaged from care will re-engage within 3 years including care received at a hospital or emergency department visit [7]. Contemporary studies that were able to determine the true status of LTF patients—which is a small number—most had transferred care to clinics closer to their home or newer clinics that provide different services; or alternatively, were alive and not engaged in care [3, 4, 7, 11, 45]. Forster et al. found a strong correlation between clinics with high LTF rates also had high rates of missing data for patient characteristics [1]. Ideally, a formal tracking system that “follows” patients when they receive care at other institutions would be an optimal way to track silent transfers; however this is still in development in most countries [3, 4, 7, 10, 48]. With these findings that most LTF patients are actually alive, our method of imputing LTF status and missing covariates, at the same time, is a cost effective method to estimate true mortality and to study risk factors for HIV.

Each of the described methods in this article has different assumptions for LTF, as well as limitations and strengths (Table 4). For complete case analysis, the loss of statistical power by automatically excluding observations that have missing information is a concern for many researchers [15, 29]. This automatic exclusion leaves room for bias depending on the types and patterns of missingness [28, 29]. Many HIV studies have found that the underlying assumption that LTF is unrelated to mortality is an incorrect assumption and thus survival estimates and associations of death to be biased and incorrectly estimated [17, 21, 25, 49]. Clinicians report that those who were LTF back in the early 2000’s were later found to be dead compared to more contemporary cohorts whose LTF participants are more likely to be alive [13, 22, 25, 50, 51].

**Table 4:** Assumptions, Limitations, Strengths and Biases between different methods of analysis

Method	Assumptions	Limitations	Strengths	Bias
Complete Case Analysis	Participants with missing data are a random sample of those intended to be observed [15, 29]	Loss of statistical power [56] Prone to bias [29]	Automatically implemented by software Common method	Might be biased if participants with missing data are different to those with complete data [15]
Survival Analysis	LTF is unrelated to mortality	Most studies found assumption to be incorrect Survival is usually overestimated	Most common method Easy to perform	
Inverse Probability Weights from Tracing	Those unsuccessfully traced have the same mortality as those successfully traced “outcomes are missing at random after accounting for available covariates” [22]	Tracing was done at the end of the 10 year follow up period on everyone Case-wise deletion if covariates are missing Tracing can be difficult and expensive Only as successful as your tracing success Loss of statistical power [56]	Common method in HIV studies Conceptually easy to understand Best employed for monotone missing data [29]	Biased estimate of effect size [56] Residual selection bias [22]
Multiple Imputation with Chained Equations	Missing are only randomly different from patients with same set of covariates	Relies on a good prediction model Susceptible to human error [29]	Use all observations Robust standard error Least biased estimates of effect size [56] Gains in precision of estimation of effects [15]	If data are not MCAR results might be biased away from the null [29]

With regards to IPW from tracing data, there are many limitations associated with this methodology. IPW from tracing techniques assume that the traced participants are a representative sample of all LTF. With this assumption in mind, a random sample of LTF participants is selected for tracing [13, 20, 21, 52–55]. In this cohort, tracing was attempted on all participants who were LTF and was performed with telephone and in-person follow up. Additionally, in this cohort, tracing was done at the end of the 10 year follow-up period, and those who were more recently lost were more likely to be found compared to those lost at the beginning of the follow-up period. Another limitation, inherent in most IPW analyses, is the non-inclusion of several observations because of automatic case-wise deletion by the analysis software due to missing data. With this in mind, estimates might be biased and a loss of statistical power might occur when utilizing this method [22, 56].

Unlike IPW, MICE is able to use all the observations in a dataset by imputing the missing values, resulting in robust results. However, it too has assumptions and is prone to limitations. One major assumption is that the risk of death among patients who are LTF is constant over time. This may not be the case as mortality is known to be highest in early periods after ART initiation and decreases over time [33, 34, 45, 57]. Additionally, MICE relies on a good prediction model and requires data to be missing at random (MAR) [29, 31]. Although MAR is difficult to ascertain, recent publications have explored the application of MICE in non-MAR situations and found that a small amount of bias might be present in the results. However, compared to the other methods, the small amount of bias that might be present is offset by the gains of using all observations present in the dataset and the robust standard errors calculated by the procedure [29, 30, 58]. Several studies have incorporated MICE as a method to estimate associations due to attrition or lost to follow up in longitudinal studies [59, 60]. Regardless of the method used, one must diligently explore patterns of missingness before performing any analyses [10, 25, 28–31]. We believe that, despite some limitations with MICE, the benefits of using all available data and the subsequent calculation of robust standard errors outweigh the limitations. Therefore, the approach of imputing both the outcome and covariates seems better than more traditional methods.

Although we describe a statical approach to approximating survival rates, implementation research is needed to determine the effectiveness and scalability of interventions to keep patients engaged in care and to return them into care [3, 44, 45, 48]. HIV programs should consider including sensitivity analyses or other methods for estimating the vital status among those categorized as lost, as traditional methods, such as CC, IPW, Kaplan Meier and Cox proportional hazards models,do not consider that patients who are lost re-engage in care. The multiple imputation method that we describe in this paper provides an estimate that is closer to the actual outcome rates. Further research is needed to test this method in other countries and HIV programs to see if it provides outcome estimates close to actual rates.

## Conclusions

In the last ten years, there has been an increase in the number of journal articles citing multiple imputation as a method used for filling in missing values or as a secondary analysis [53, 61, 62]. MICE might be a cost efficient mathematical alternative that can be employed in resource limited settings such as Haiti to impute outcome status estimates for program evaluation to estimate survival. However, data should be evaluated for patterns of missingness. Currently, MICE is underutilized in public health research—especially of HIV-infected cohorts. Because the benefits of MICE outweigh the potential for erroneous use, we encourage the use of MICE among our HIV research colleagues.

## Additional file

Additional file 1: Data analysis using R is a supplementary file that describes how to download the free statistical software package R and R studio. It also includes the names of the R packages used for this analysis and various websites that one could consult for help using R. (DOCX 12 kb)

## Abbreviations

95% CI: 95% Confidence Interval
ART: Antiretroviral Therapy
CC: Complete Case
CD4: CD4+ cells
GHESKIO: Haitian Group for the Study of Kaposi’s Sarcoma and Opportunistic Infections
HIV: Human Immunodeficiency Virus
IPW: Inverse Probability Weight
LTF: Lost to Follow Up
MAR: Missing at Random
MICE: Multiple Imputation with Chained Equations
OR: Odds Ratio
RR: Risk Ratio
WHO: World Health Organization

## References

[1] Forster M, Bailey C, Brinkhof MWG, Graber C, Boulle A, Spohr M (2008). Electronic medical record systems, data quality and loss to follow-up: survey of antiretroviral therapy programmes in resource-limited settings. Bull World Health Organ.

[2] Lambdin BH, Micek MA, Koepsell TD, Hughes JP, Sherr K, Pfeiffer J (2012). An assessment of the accuracy and availability of data in electronic patient tracking systems for patients receiving HIV treatment in Central Mozambique. BMC Health Serv Res.

[3] McNairy ML, Joseph P, Unterbrink M, Galbaud S, Mathon J-E, Rivera V (2017). Outcomes after antiretroviral therapy during the expansion of HIV services in Haiti. PLoS One.

[4] Wolff MJ, Giganti MJ, Cortes CP, Cahn P, Grinsztejn B, Pape JW (2017). A decade of HAART in Latin America: long term outcomes among the first wave of HIV patients to receive combination therapy. PLoS One.

[5] Carriquiry G, Fink V, Koethe JR, Giganti MJ, Jayathilake K, Blevins M (2015). Mortality and loss to follow-up among HIV-infected persons on long-term antiretroviral therapy in Latin America and the Caribbean. J Int AIDS Soc.

[6] Farahani M, Vable A, Lebelonyane R, Seipone K, Anderson M, Avalos A (2014). Outcomes of the Botswana national HIV/AIDS treatment programme from 2002 to 2010: a longitudinal analysis. Lancet Glob Heal.

[7] Kaplan SR, Oosthuizen C, Stinson K, Little F, Euvrard J, Schomaker M (2017). Contemporary disengagement from antiretroviral therapy in Khayelitsha South Africa: A cohort study. PLOS Med.

[8] Mberi MN, Kuonza LR, Dube NM, Nattey C, Manda S, Summers R (2015). Determinants of loss to follow-up in patients on antiretroviral treatment, South Africa, 2004–2012: a cohort study. BMC Health Serv Res.

[9] Sowah LA, Turenne FV, Buchwald UK, Delva G, Mesidor RN, Dessaigne CG (2014). Influence of transportation cost on long-term retention in clinic for HIV patients in rural Haiti. JAIDS J Acquir Immune Defic Syndr.

[10] Puttkammer NH, Zeliadt SB, Baseman JG, Destiné R, Wysler Domerçant J, Labbé Coq NR (2014). Patient attrition from the HIV antiretroviral therapy program at two hospitals in Haiti. Rev Panam Salud Publica.

[11] Gloyd S, Wagenaar BH, Woelk GB, Kalibala S. Opportunities and challenges in conducting secondary analysis of HIV programmes using data from routine health information systems and personal health information. J Int AIDS Soc. 2016;19(5 4). 10.7448/IAS.19.5.20847. 10.7448/IAS.19.5.20847 PMC4956739 27443274

[12] Maskew M, MacPhail P, Menezes C, Rubel D (2007). Lost to follow up: contributing factors and challenges in south African patients on antiretroviral therapy. S Afr Med J.

[13] Tweya H, Feldacker C, Estill J, Jahn A, Ng’ambi W, Ben-Smith A (2013). Are they really lost? “True” status and reasons for treatment discontinuation among HIV infected patients on antiretroviral therapy considered lost to follow up in urban Malawi. PLoS One.

[14] Geng E. H., Glidden D. V., Bangsberg D. R., Bwana M. B., Musinguzi N., Nash D., Metcalfe J. Z., Yiannoutsos C. T., Martin J. N., Petersen M. L. (2012). A Causal Framework for Understanding the Effect of Losses to Follow-up on Epidemiologic Analyses in Clinic-based Cohorts: The Case of HIV-infected Patients on Antiretroviral Therapy in Africa. American Journal of Epidemiology.

[15] Karahalios A, Baglietto L, Carlin JB, English DR, Simpson JA (2012). A review of the reporting and handling of missing data in cohort studies with repeated assessment of exposure measures. BMC Med Res Methodol.

[16] Kenward M G, Molenberghs G (1999). Parametric models for incomplete continuous and categorical longitudinal data. Statistical Methods in Medical Research.

[17] Kurth T, Walker AM, Glynn RJ, Chan KA, Gaziano JM, Berger K (2006). Results of multivariable logistic regression, propensity matching, propensity adjustment, and propensity-based weighting under conditions of nonuniform effect. Am J Epidemiol.

[18] Lippman SA, Shade SB, Hubbard AE (2010). Inverse probability weighting in STI/HIV prevention research: methods for evaluating social and community interventions. Sex Transm Dis.

[19] Buchanan AL, Hudgens MG, Cole SR, Lau B, Adimora AA (2014). Worth the weight: using inverse probability weighted cox models in AIDS research. AIDS Res Hum Retrovir.

[20] Van Cutsem G, Ford N, Hildebrand K, Goemaere E, Mathee S, Abrahams M (2011). Correcting for mortality among patients lost to follow up on antiretroviral therapy in South Africa: a cohort analysis. PLoS One.

[21] Henriques J, Pujades-Rodriguez M, McGuire M, Szumilin E, Iwaz J, Etard J-F (2012). Comparison of methods to correct survival estimates and survival regression analysis on a large HIV African cohort. PLoS One.

[22] Geng EH, Glidden DV, Bangsberg DR, Bwana MB, Musinguzi N, Nash D (2012). A causal framework for understanding the effect of losses to follow-up on epidemiologic analyses in clinic-based cohorts: the case of HIV-infected patients on antiretroviral therapy in Africa. Am J Epidemiol.

[23] Goel MK, Khanna P, Kishore J (2010). Understanding survival analysis: Kaplan-Meier estimate. Int J Ayurveda Res.

[24] Brinkhof MWG, Pujades-Rodriguez M, Egger M (2009). Mortality of patients lost to follow-up in antiretroviral treatment Programmes in resource-limited settings: systematic review and meta-analysis. PLoS One.

[25] Geng EH, Odeny TA, Lyamuya RE, Nakiwogga-Muwanga A, Diero L, Bwana M (2015). Estimation of mortality among HIV-infected people on antiretroviral treatment in East Africa: a sampling based approach in an observational, multisite, cohort study. Lancet HIV.

[26] Plutzer K, Mejia GC, Spencer AJ, Keirse MJNC (2010). Dealing with missing outcomes: lessons from a randomized trial of a prenatal intervention to prevent early childhood caries. Open Dent J.

[27] Tanski SE, McClure AC, Li Z, Jackson K, Morgenstern M, Li Z (2015). Cued recall of alcohol advertising on television and underage drinking behavior. JAMA Pediatr.

[28] Knol MJ, Janssen KJM, Donders ART, Egberts ACG, Heerdink ER, Grobbee DE (2010). Unpredictable bias when using the missing indicator method or complete case analysis for missing confounder values: an empirical example. J Clin Epidemiol.

[29] White IR, Carlin JB (2010). Bias and efficiency of multiple imputation compared with complete-case analysis for missing covariate values. Stat Med.

[30] White IR, Royston P, Wood AM (2011). Multiple imputation using chained equations: issues and guidance for practice. Stat Med.

[31] Hedden SL, Woolson RF, Carter RE, Palesch Y, Upadhyaya HP, Malcolm RJ (2009). The impact of loss to follow-up on hypothesis tests of the treatment effect for several statistical methods in substance abuse clinical trials. J Subst Abus Treat.

[32] Leger Paul, Charles Macarthur, Severe Patrice, Riviere Cynthia, Pape Jean William, Fitzgerald Daniel W. (2009). 5-Year Survival of Patients with AIDS Receiving Antiretroviral Therapy in Haiti. New England Journal of Medicine.

[33] Severe Patrice, Leger Paul, Charles Macarthur, Noel Francine, Bonhomme Gerry, Bois Gyrlande, George Erik, Kenel-Pierre Stefan, Wright Peter F., Gulick Roy, Johnson Warren D., Pape Jean William, Fitzgerald Daniel W. (2005). Antiretroviral Therapy in a Thousand Patients with AIDS in Haiti. New England Journal of Medicine.

[34] Rodwell L, Lee KJ, Romaniuk H, Carlin JB. Comparison of methods for imputing limited-range variables: a simulation study. BMC Med Res Methodol. 2014;14:57. 10.1186/1471-2288-14-57. 10.1186/1471-2288-14-57 PMC4021274 24766825

[35] Allison P. Imputation by Predictive Mean Matching: Promise &amp; Peril | Statistical Horizons. March 5. 2015. https://statisticalhorizons.com/predictive-mean-matching. Accessed 13 Jan 2018.

[36] Vink Gerko, Frank Laurence E., Pannekoek Jeroen, van Buuren Stef (2014). Predictive mean matching imputation of semicontinuous variables. Statistica Neerlandica.

[37] Morris TP, White IR, Royston P. Tuning multiple imputation by predictive mean matching and local residual draws. BMC Med Res Methodol. 2014;14:75. 10.1186/1471-2288-14-75. 10.1186/1471-2288-14-75 PMC4051964 24903709

[38] Eddings W, Marchenko Y, Eddings W, Marchenko Y. Diagnostics for multiple imputation in Stata. Stata J 2012;12:353–367. http://econpapers.repec.org/article/tsjstataj/v_3a12_3ay_3a2012_3ai_3a3_3ap_3a353-367.htm. Accessed 8 Sept 2017.

[39] Recursive Partitioning and Regression Trees [R package rpart version 4.1–11]. https://cran.r-project.org/web/packages/rpart/index.html. Accessed 15 Nov 2017.

[40] Harrell FE, E. F. Regression modeling strategies : with applications to linear models, logistic regression, and survival analysis. Springer; 2001. http://dl.acm.org/citation.cfm?id=1196963. Accessed 22 Sept 2017.

[41] Hastie, Trevor, Tibshirani, Robert, Friedman J. The Elements of Statistical Learning: Data Mining, Inference, and Prediction. 2nd ed. Stanford: Springer; 2016.

[42] Rubin DB. Multiple imputation for nonresponse in surveys: Wiley-Interscience; 2004.

[43] Hippel PT von. Regression with Missing Ys: An Improved Strategy for Analyzing Multiply Imputed Data. Sociological Methodology. 37:83–117. 10.2307/20451132.

[44] Pierre Samuel, Jannat-Khah Deanna, Fitzgerald Daniel W., Pape Jean, McNairy Margaret L. (2016). 10-Year Survival of Patients with AIDS Receiving Antiretroviral Therapy in Haiti. New England Journal of Medicine.

[45] Noel Edva, Esperance Morgan, Mclaughlin Megan, Bertrand Rachel, Devieux Jessy, Severe Patrice, Decome Diessy, Marcelin Adias, Nicotera Janet, Delcher Chris, Griswold Mark, Meredith Genevive, Pape Jean William, Koenig Serena P. (2013). Attrition From HIV Testing to Antiretroviral Therapy Initiation Among Patients Newly Diagnosed With HIV in Haiti. JAIDS Journal of Acquired Immune Deficiency Syndromes.

[46] Pierre Samuel, Jannat-Khah Deanna, Fitzgerald Daniel W., Pape Jean, McNairy Margaret L. (2016). 10-Year Survival of Patients with AIDS Receiving Antiretroviral Therapy in Haiti. New England Journal of Medicine.

[47] Coria Alexandra, Noel Francine, Bonhomme Jerry, Rouzier Vanessa, Perodin Christian, Marcelin Adias, Li Zhongze, Tosteson Tor D., Deschamps Marie-Marcelle, Wright Peter F., Pape Jean W. (2012). Consideration of Postpartum Management in HIV-Positive Haitian Women. JAIDS Journal of Acquired Immune Deficiency Syndromes.

[48] Hennessey KA, Leger TD, Rivera VR, Marcelin A, McNairy ML, Guiteau C (2017). Retention in care among patients with early HIV disease in Haiti. J Int Assoc Provid AIDS Care.

[49] Falcaro M, Nur U, Rachet B, Carpenter JR (2015). Estimating excess Hazard ratios and net survival when covariate data are missing. Epidemiology.

[50] Wubshet M, Berhane Y, Worku A, Kebede Y (2013). Death and seeking alternative therapy largely accounted for lost to follow-up of patients on ART in Northwest Ethiopia: a community tracking survey. PLoS One.

[51] Caluwaerts C, Maendaenda R, Maldonado F, Biot M, Ford N, Chu K (2009). Risk factors and true outcomes for lost to follow-up individuals in an antiretroviral treatment programme in Tete Mozambique. Int Health.

[52] Reidy W, Agarwal M, Lamb M, Hawken M, Chege D, Elul B (2014). Loss to follow-up: determining outcomes for adults enrolled in HIV Services in Kenya.

[53] Schomaker M, Gsponer T, Estill J, Fox M, Boulle A (2014). Non-ignorable loss to follow-up: correcting mortality estimates based on additional outcome ascertainment. Stat Med.

[54] Geng Elvin H, Odeny Thomas A, Lyamuya Rita E, Nakiwogga-Muwanga Alice, Diero Lameck, Bwana Mwebesa, Muyindike Winnie, Braitstein Paula, Somi Geoffrey R, Kambugu Andrew, Bukusi Elizabeth A, Wenger Megan, Wools-Kaloustian Kara K, Glidden David V, Yiannoutsos Constantin T, Martin Jeffrey N (2015). Estimation of mortality among HIV-infected people on antiretroviral treatment in east Africa: a sampling based approach in an observational, multisite, cohort study. The Lancet HIV.

[55] Geng EH, Glidden DV, Bwana MB, Musinguzi N, Emenyonu N, Muyindike W (2011). Retention in care and connection to care among HIV-infected patients on antiretroviral therapy in Africa: estimation via a sampling-based approach. PLoS One.

[56] Witkiewitz K, Falk DE, Kranzler HR, Litten RZ, Hallgren KA, O’Malley SS (2014). Methods to analyze treatment effects in the presence of missing data for a continuous heavy drinking outcome measure when participants drop out from treatment in alcohol clinical trials. Alcohol Clin Exp Res.

[57] Lawn SD, Campbell L, Kaplan R, Boulle A, Cornell M, Kerschberger B (2011). Time to initiation of antiretroviral therapy among patients with HIV-associated tuberculosis in Cape Town, South Africa. JAIDS J Acquir Immune Defic Syndr.

[58] White IR, Royston P (2009). Imputing missing covariate values for the cox model. Stat Med.

[59] Biering K, Hjollund NH, Frydenberg M (2015). Using multiple imputation to deal with missing data and attrition in longitudinal studies with repeated measures of patient-reported outcomes. Clin Epidemiol.

[60] McCaul KA, Almeida OP, Norman PE, Yeap BB, Hankey GJ, Golledge J (2015). How Many Older People Are Frail? Using Multiple Imputation to Investigate Frailty in the Population. J Am Med Dir Assoc.

[61] Mackinnon A (2010). The use and reporting of multiple imputation in medical research - a review. J Intern Med.

[62] Fatti G, Meintjes G, Shea J, Eley B, Grimwood A (2012). Improved survival and antiretroviral treatment outcomes in adults receiving community-based adherence support: 5-year results from a multicentre cohort study in South Africa. J Acquir Immune Defic Syndr.

