# Supplementary material for: Treating loss-to-follow-up as a missing data problem: a case study using a longitudinal cohort of HIV-infected patients in Haiti
Source: BMC Public Health. 2018 Nov 19;18:1269. doi: 10.1186/s12889-018-6115-0 (PMC6245624; doi:10.1186/s12889-018-6115-0)
Supplement: Supplementary file 1 — Data analysis using R is a supplementary file that describes how to download the free statistical software package R and R studio. It also includes the names of the R packages used for this analysis and various websites that one could consult for help using R. (DOCX 12 kb) [file 12889_2018_6115_MOESM1_ESM.docx]

Data Analysis using R

For this manuscript, we utilized the free statistical software R and interface R studio. Both are available for download free of charge.

<https://www.r-project.org/>

<https://www.rstudio.com/>

The R packages that were used for this analysis were Hmisc and Rms. These two packages were written by Frank Harrell and have commands available to perform multiple imputation, linear and logistic regression and survival analysis.

Help with troubleshooting commands and analyses can be accessed through various websites including the following:

Stats.stackexchange.com

<http://www.fharrell.com/>

References:

Harrell FE, E. F. Regression modeling strategies : with applications to linear models, logistic regression, and survival analysis. Springer; 2001. http://dl.acm.org/citation.cfm?id=1196963. Accessed 22 Sep 2017.
